# Effect of regional block technique on postoperative high-grade complications according to Clavien-Dindo classification in elderly patients with thoracic and abdominal cancer: a retrospective propensity score matching analysis

**DOI:** 10.3389/fonc.2023.1305329

**Published:** 2023-12-27

**Authors:** Weisi Ding, Yunpeng Zhang, Huixin Liu, Tianxin Zhou, Wanlu Zhao, Yi Feng, Haiyan An

**Affiliations:** ^1^Department of Anesthesiology, Peking University People’s Hospital, Beijing, China; ^2^Department of Clinical Epidemiology and Biostatistics, Peking University People's Hospital, Beijing, China

**Keywords:** regional block, old age, postoperative complications, pain, Clavien-Dindo classification, cancer

## Abstract

**Background:**

Postoperative complications have an influence on postoperative rehabilitation, length of hospital stay and hospitalization expenses in elderly patients, especially those with higher Clavien-Dindo (C-D) classification. Patients with cancers often experience more serious postoperative complications after surgery. Different anesthesia methods can affect the postoperative outcomes of cancer patients. Regional block techniques have been recommended in guidelines for enhanced recovery after surgery. However, the relationship between regional blocks and high-grade postoperative complications remains unclear, thus, the study explored the relationship between regional block techniques and high-grade postoperative complications graded by C-D classification in elderly patients with thoracic and abdominal cancer.

**Method:**

Retrospective enrollment of eligible elderly patients admitted to Peking University People’s Hospital between January 2018 and March 2022 was conducted. Propensity score matching (PSM) and univariate and multivariate regression analyses were used to analyze the potential benefits of regional blocks for elderly patients in real world practice.

**Results:**

A total of 2769 patients were enrolled in this study, including 568 who underwent colorectal resection, 2201 who underwent video-assisted thoracoscopic pneumonectomy. Among them, 2033 patients received regional block, while 736 patients did not. Statistical analysis indicated that regional blocks could reduce the incidence of postoperative complications of C-D classification Grade II or higher, with an Odds ratio (OR) of 0.742, 95% Confidence interval (CI) (0.552 to 0.996) (*P* = 0.047).

**Conclusion:**

Regional block is associated with a reduction in the occurrence of postoperative complications graded by C-D classification in elderly patients with thoracic and abdominal cancer. The application of regional blocks can lower the risk of high-risk complications and mortality.

## Introduction

The postoperative outcome of cancer patients is often the focus of attention. Different anesthesia methods can affect the postoperative outcome of cancer patients (1).Poorly controlled perioperative pain has been shown to associate with increased morbidity, impaired quality of life, longer hospital stays, more opioid use, and higher healthcare costs (2). Systemic opioid analgesics has been used to provide relief from severe trauma-related pain but their use is hampered by serious risks such as respiratory depression, opioid-use disorder, and potentially fatal overdose (3). The more the patients take and the longer the patient takes opioids for, the higher the risk for becoming emotionally and physically dependent on them. Another common type of regional anesthesia is the peripheral regional block (PNB), which is produced by injections made with great exactness near a cluster of nerves to numb the appropriate area of the body extremity (arm, leg, trunk) that requires surgery. Studies have consistently found that patients receiving PNBs experienced superior pain control and less opioid consumption in a wide range of operational procedures, including colorectal surgery, thoracic surgery (4–6). Obviously, PNBs provide a safe and effective way to improve pain management and reduce opioid consumption (7). It has been recommended by Guidelines for Perioperative Care in Elective Colorectal Surgery to accelerate recovery after surgery (8). Such Clinical recommendations are often based on evidence derived from meta-analysis of randomized controlled trials (RCTS) but their interpretation is often hindered by the fact that they do not always consider current clinical relevance (9). Recently, a few studies have found that the use of regional blocks can reduce the incidence of postoperative complications in patients with real data (10), however, the findings are more limited to orthopedic surgery and do not grade the severity of complications in them (11, 12). The same complications may have different levels of severity with different levels of management. The severity of postoperative complications in patients is often graded during surgery according to the Clavien-Dindo (C-D) classification, which grading complications by postoperative management measures (13). Consistent and comparative regional anesthesia outcome data are still lacking, and no study has systematically examined the effects of PNBs on the occurrence of postoperative complications graded by the C-D classification in elder patients with cancer (14). Another challenge lies in the management of perioperative pain in elderly patients. Not only the number of procedures performed on patients over 65 years of age increases significantly, but also older patients are at increased risk of adverse postoperative outcomes (15, 16). In prospective cohort study conducted in the United States, patients aged 80 and above showed significantly higher 30-day all-cause mortality than younger patients (17). As a part of multimodal analgesia, PNBs are preferred for anesthesia in elderly patients to promote rapid recovery after surgery. To the authors’ knowledge, studies evaluating the effect of PNBs on all postoperative complications graded by the C-D classification in elderly patients with cancer have not been performed. The aim of the present study was to investigate the relationship of PNBs with high-grade complications in older patients with thoracic and abdominal cancer using real-world data. Our findings provide expanded insights to guide clinical practice and optimize patient care.

## Methods

### Ethical considerations

This single-center retrospective study was conducted in accordance with the Declaration of Helsinki at Peking University People’s Hospital between 1 January 2018 and 31 March 2022. Ethical approval for this study (Approval No. 2022PHB159-001) was provided by the Medical Ethics Committee of Peking University People’s Hospital, Beijing, China (Chairperson: Dr. Xueguang Zhu) in 2022.

### Inclusion and exclusion criteria

Patients aged 65 years or more were enrolled in this study. Inclusion criteria was scheduled for elective surgery including radical colorectal resection and thoracoscopic pneumonectomy. Patients who were admitted to the intensive care unit (ICU) before the surgery, those who underwent a second or more operation during this admission, or those with missing or incomplete prognostic records were excluded from this study.

### Anesthetic procedure

All patients were divided into two groups, the regional block group (RB) was anesthetized with general anesthesia and regional block, and the general anesthesia group (GA) was only anesthetized with general anesthesia. Preoperative regional block was carried out by an experienced anesthesiologist in a dedicated room equipped with professional operating equipment (ultrasound, nerve stimulator, etc.) according to the patient’s surgical site. Transversus abdominis plane block (TAPB) was used for colorectal resection, paravertebral block was provided for thoracoscopic pneumonectomy. All regional block procedures were performed under ultrasound guidance. Low concentrations of ropivacaine were used as local anesthetics for all regional blocks.

All patients underwent general anesthesia with intravenous administration of propofol (1.5 to 2.5 mg/Kg), sufentanil (0.3 µg/Kg) and rocuronium (0.6 mg/Kg). Anesthesia was maintained by inhalation of sevoflurane or desflurane and continuous infusion of propofol and remifentanil to keep the bispectral index (BIS) between 40 and 60. After surgery, the patients were extubated in post-anesthesia care unit (PACU) or in operation room or transferred to the ICU with endotracheal intubation. All extubations were followed by consciousness, respiratory and circulatory stability, and recovery of muscle strength, and the patient was subsequently returned to the ward.

Moreover, during all surgeries, vasopressor agents can be empirically used by the anesthesiologist according to the patient’s initial and intraoperative blood pressure.

### Postoperative complication assessment

The C-D classification and comprehensive complication index (CCI) were used as the primary outcome to evaluate postoperative complications. The C-D classification is a standardized system to report postoperative morbidity by rating any deviation from the normal postoperative course in 7 grades (I, II, IIIa, IIIb, IVa, IVb and V) (13). Grade I complications are usually mild but Grade II and higher complications are more significant. The CCI is a widely-used tool to assess patients’ overall morbidity after an intervention. It based on the complication grading by the C-D classification and calculated as the sum of all complications that were weighted for their severity by patients and physicians, with the final formula yielding a score than rages from 0 (no complication) to 100 (death) (18, 19). In this study, postoperative complications were graded according to the official website (https://www.assessurgery.com/clavien-dindo-classification/) (**Table 1**), and the CCI was calculated using the CCI Calculator (AssesSurgery GmbH c/o GHM Partners AG Poststrasse 24 6300 Zug). To further analyze the association of anesthetic procedure with postoperative complication, additional data were collected and analyzed as the secondary outcome, including postoperative length of stay (LOS) and hospitalization expense (ten thousand RMB).

**Table 1:** The Clavien-Dindo Classification.

Grades	Definition
Grade I	Any deviation from the normal postoperative course without the need for pharmacological treatment or surgical, endoscopic and radiological interventions. Allowed therapeutic regimens are: drugs as antiemetics, antipyretics, analgetics, diuretics and electrolytes and physiotherapy. This grade also includes wound infections opened at the bedside.
Grade II	Requiring pharmacological treatment with drugs other than such allowed for grade I complications. Blood transfusionsand total parenteral nutritionare also included.
Grade III - IIIa - IIIb	Requiring surgical, endoscopic or radiological intervention Intervention not under general anesthesia Intervention under general anesthesia
Grade IV - Iva - IVb	Life-threatening complication (including CNS complications)* requiring IC/ICU-management single organ dysfunction (including dialysis) multiorgandysfunction
Grade V	Death of a patient

*brain hemorrhage, ischemic stroke, subarrachnoidalbleeding, but excluding transient ischemic attacks (TIA); IC, Intermediate care; ICU, Intensive care unit.

Quoted from https://www.assessurgery.com/ clavien-dindo-classification/.

### Data collection

Data collection included patients’ characteristics [gender, age, body mass index (BMI), ASA classification], previous medical history (hypertension, diabetes, coronary heart disease or other types of heart diseases, chronic lung disease, liver failure, renal insufficiency, venous thromboembolism, coagulation dysfunction, immune system disorders, hypoproteinemia, anemia, and hyponatremia) and preoperative medications (antiplatelet or anticoagulant therapy, chemotherapy). Preoperative hypoproteinemia, anemia and hyponatremia were determined based on the last laboratory examination before surgery. Anemia was defined as a hemoglobin concentration of less than 13 g/L in men and 12 g/L in women. Hypoproteinemia was defined as albumin of less than 30 g/L or total protein of less than 60 g/L. Hyponatremia was defined as a serum sodium concentration of less than 135 mmol/L. The details about the operation were also recorded such as duration, intraoperative use of vasopressor, application of regional block.

### Statistical analysis

Statistical analyses were performed using SPSS version 26.0 (SPSS Inc, Chicago, IL, USA). Continuous data were expressed as mean ± standard deviation (SD), and categorical data were expressed as frequency and percent. The differences between continuous variables with from Gaussian distribution were compared using Student’s t-test. Otherwise, the non-parametric Mann-Whitney U test was used. Categorical data were compared using Pearson’s chi-square test or Fisher’s exact test. 1:1 propensity score matching was performed with duration, ASA classification, preoperative venous thromboembolism and chemotherapy. Univariable and multivariable logistic regression were used to identify the factors associated with categorical outcome measures, univariable and multivariable linear regression were used to identify the factors associated with continuous outcome measures. A *P* value of less than 0.2 and factors thought to have influenced in the results were admitted to the multivariable logistic regression. A *P* value of less than 0.05 was considered statistically significant.

## Results

### Patient characteristics

**Figure 1:**
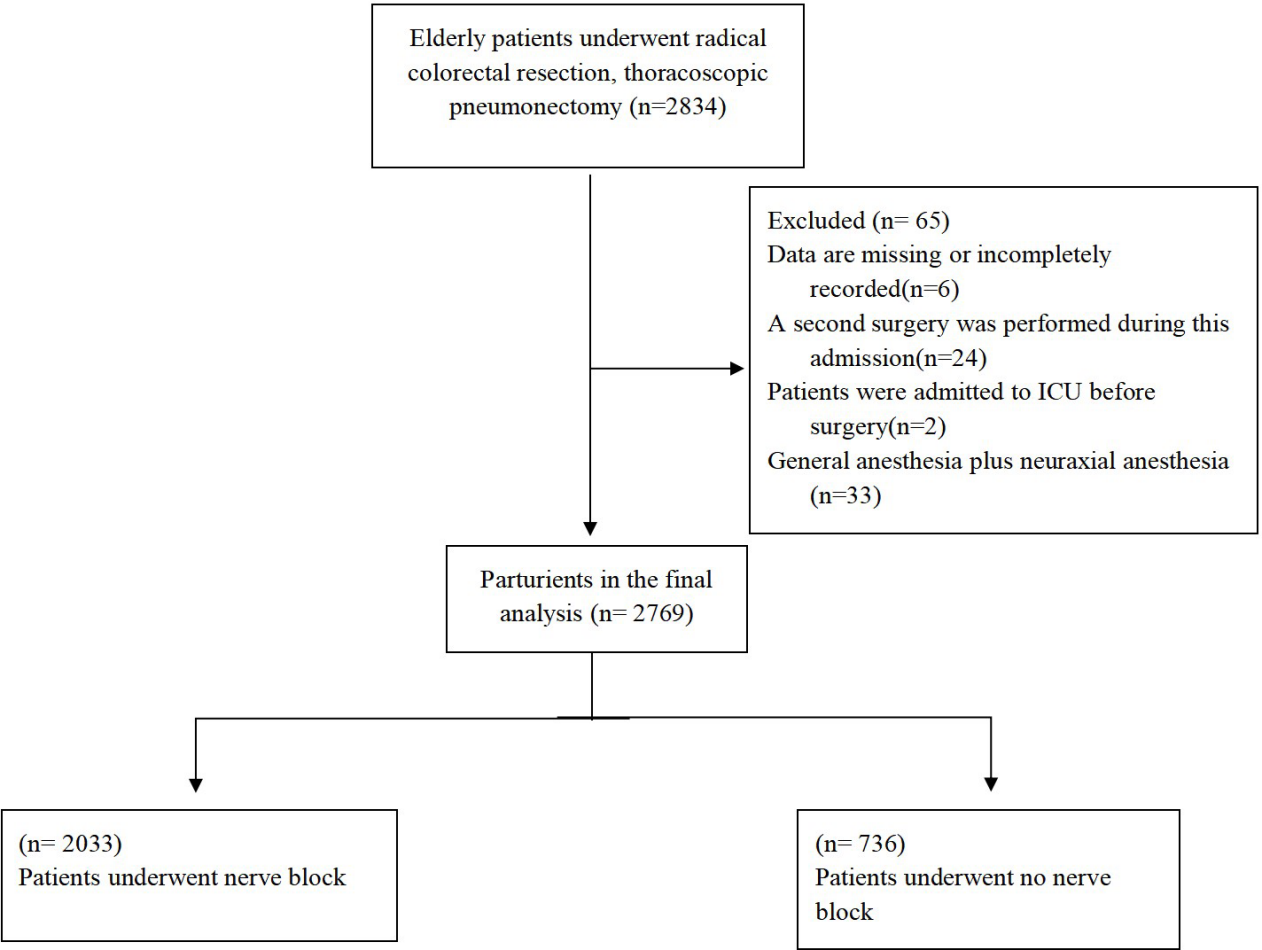
Quoted from https://www.assessurgery.com/clavien-dindo-classification/.

As shown in **Figure 1**, a total of 2769 eligible subjects who underwent elective surgery were enrolled in the study. All eligible subjects were divided into regional block (RB) group (2033 patients, 73.4%) and general anesthesia (GA) group (736 patients, 26.6%). There were statistically significant differences between the two groups in gender(*P*=0.022), ASA classification(*P*<0.001), type of surgery(*P*<0.001), duration(*P*<0.001), use vasopressor during the surgery(*P*=0.008), preoperative diabetes(*P*=0.048), chronic lung disease(*P*=0.010),venous thromboembolism(*P*=0.001), hypoproteinemia(*P*=0.018), anemia(*P*<0.001), hyponatremia(*P*=0.023), chemotherapy(*P*=0.003) (**Table 2**). After matching, all eligible subjects were divided into RB group (712 patients, 50.0%) and GA group (712 patients, 50.0%). There were no statistically significant differences between the two groups other than type of surgery(*P*<0.001), preoperative anemia(*P*=0.003) and antiplatelet or anticoagulant therapy(*P*=0.025).

**Table 2:** Comparison of patient demographics, intraoperative findings and preoperative history between groups.

	Unmatched			Matched		
	RB (2033)	GA (736)	P value*	RB (712)	GA (712)	P value*
Patient characteristics						
Age, mean (SD)	71 (5)	72 (6)	0.065	71 (5)	72 (6)	0.635
Females, n (%)	1034 (50.9)	398 (54.1)	**0.022**	323 (45.4)	325 (45.6)	0.915
BMI, n (%)			0.063			0.069
* > 28 Kg/m^2^*	242 (11.9)	69 (9.4)		85 (11.9)	64 (9.0)	
* ≤28 Kg/m^2^*	1791 (88.1)	667 (90.6)		627 (88.1)	648 (91.0)	
ASA, n (%)			**<0.001**			1.000
* ≥ Grade III*	330 (16.2)	204 (27.7)		191 (26.8)	191 (26.8)	
* < Grade III*	1703 (83.8)	532 (72.3)		521 (73.2)	521 (73.2)	
Surgery and anesthesia, n (%)						
Type of surgery			**<0.001**			**<0.001**
* Radical colorectal resection*	200 (9.8)	368 (50.0)		131 (18.4)	344 (48.3)	
* Thoracoscopic pneumonectomy*	1833 (90.2)	368 (50.0)		581 (81.6)	368 (51.7)	
Duration			**<0.001**			0.955
* > 3 h*	326 (16.0)	266 (36.1)		241 (33.8)	242 (34.0)	
* ≤ 3 h*	1707 (84.0)	470 (63.9)		471 (66.2)	470 (66.0)	
Use vasopressor during operation, n (%)	1276(62.8)	502 (68.2)	**0.008**	461 (64.7)	483 (67.8)	0.217
Previous history, n (%)						
Hypertension	982 (48.3)	356 (48.4)	0.975	326 (45.8)	345 (48.5)	0.313
Diabetes	405 (19.9)	172 (23.4)	**0.048**	145 (20.4)	163 (22.9)	0.247
Coronary heart disease	299 (14.7)	98 (13.3)	0.356	118 (16.6)	93 (13.1)	0.062
Other heart diseases	188 (9.2)	70 (9.5)	0.833	69 (9.7)	69 (9.7)	1.000
Chronic lung disease	50 (2.5)	32 (4.3)	**0.010**	19 (2.7)	30 (4.2)	0.110
Liver failure	47 (2.3)	16 (2.2)	0.830	16 (2.2)	16 (2.2)	1.000
Renal insufficiency	40 (2.0)	15 (2.0)	0.906	15 (2.1)	14 (2.0)	0.851
Venous thromboembolism	7 (0.34)	11 (1.5)	**0.001**	6 (0.84)	5 (0.70)	0.762
Coagulation dysfunction	9 (0.44)	3 (0.41)	0.901	4 (0.56)	3 (0.42)	0.705
Immune system disorder	17 (0.84)	2 (0.27)	0.112	5 (0.70)	2 (0.28)	0.452
Hypoproteinemia	189 (9.3)	91 (12.4)	**0.018**	82 (11.5)	85 (11.9)	0.805
Anemia	405 (19.9)	238 (32.3)	**<0.001**	176 (24.7)	226 (31.7)	**0.003**
Hyponatremia	27 (1.3)	19 (2.6)	**0.023**	17 (2.4)	18 (2.5)	0.864
Antiplatelet or anticoagulant therapy	327 (16.1)	106 (14.4)	0.282	130 (18.3)	99 (13.9)	**0.025**
Chemotherapy	59(2.9)	39 (5.3)	**0.003**	19 (2.7)	19 (2.7)	1.000

*Fisher’s exact test was used for dichotomous and categorical variables; Mann-Whitney (Wilcoxon rank-sum) test was used for continuous variables; Propensity score matching was performed based on ASA classification, duration of surgery, venous thromboembolism, and preoperative chemotherapy.

RB, regional block; GA, general anesthesia; BMI, body mass index; ASA, American Society of Anesthesiologists.P value of less than 0.05 was considered statistically significant.

### Postoperative complications graded by C-D classification

Before matching, 140(6.9%) patients had no complication in RB group, and 22(3.0%) patients had no complication in GA group. Among those receiving a regional block, 15 (0.74% of all patients) of the complications were rated as Grade III, including 10 (0.49% of all) as Grade IIIa(*P*<0.001) and 5 (0.25% of all) as Grade IIIb(*P*=0.004). Besides, 8 (0.39% of all patients) of the complications were rated as Grade IV, including 2 (0.10% of all) as Grade IVa(*P*=1.000) and 6 (0.30% of all) as Grade IVb(*P*=1.000). 3(0.15%) patients experienced C-D classification Grade V complication in those who received a regional block(*P*=0.570). After matching, 34(4.8%) patients had no complication in RB group, and 22(3.1%) patients had no complication in GA group (*P*=0.102). 460(64.6%) patients had C-D classification Grade I with regional block and 347(48.7) patients had C-D classification Grade I without regional block (*P*<0.001). 202(28.4%) patients had C-D classification Grade II with regional block and 319(44.8) patients had C-D classification Grade II without regional block (*P*<0.001). 7 (0.98% of all patients) of the complications were rated as Grade III, including 5 (0.70% of all) as Grade IIIa (*P*=0.058) and 2 (0.28% of all) as Grade IIIb (*P*=0.057), 6 (0.84% of all patients) of the complications were rated as Grade IV, including 1 (0.14% of all) as Grade IVa (*P*=1.000) and 5 (0.70% of all) as Grade IVb (*P*=0.452) and 3(0.42%) patients had C-D classification Grade V in those who received a regional block. (Get more specific information in **Table 3**).

**Table 3:** Comparison of C-D classification, CCI and postoperative length of stay, total cost between two groups.

	Unmatched			Matched		
	RB (2033)	GA (736)	P value*	RB (712)	GA (712)	P value*
Clavien-Dindo classification						
No complication, n (%)	140 (6.9)	22 (3.0)	**<0.001**	34 (4.8)	22 (3.1)	0.102
I, n (%)	1429 (70.3)	348 (47.3)	**<0.001**	460 (64.6)	347 (48.7)	**<0.001**
II, n (%)	439 (21.6)	340 (46.2)	**<0.001**	202 (28.4)	319 (44.8)	**<0.001**
IIIa, n (%)	10 (0.49)	14 (1.9)	**<0.001**	5 (0.70)	13 (1.8)	0.058
IIIb, n (%)	5 (0.25)	9 (1.2)	**0.004**	2 (0.28)	8 (1.1)	0.057
IVa, n (%)	2 (0.10)	1 (0.14)	1.000	1 (0.14)	1 (0.14)	1.000
IVb, n (%)	6 (0.30)	2 (0.10)	1.000	5 (0.70)	2 (0.28)	0.452
V, n (%)	3 (0.15)	0 (0)	0.570	3 (0.42)	0 (0)	0.249
≥Grade II, n (%)	462 (22.7)	371 (50.4)	**0.007**	216 (30.3)	348 (48.9)	**<0.001**
≥Grade III, n (%)	33 (1.6)	40 (5.4)	0.270	19 (2.7)	38 (5.3)	**0.010**
CCI, %, mean(SD)	14.0 (9.7)	20.6 (11.9)	**0.036**	16.2 (11.9)	20.2 (11.7)	**<0.001**
postoperative length of stay, mean(SD)	5 (4)	8 (10)	0.133	6 (4)	8 (9)	**<0.001**
Total cost^#^, mean(SD)	7.8 (3.8)	10.0 (18.2)	0.397	8.6 (4.7)	9.9 (18.4)	**0.019**

*Fisher’s exact test was used for dichotomous and categorical variables; Mann-Whitney (Wilcoxon rank-sum) test was used for continuous variables.

^#^Ten thousand RMB.

RB, regional block; GA, general anesthesia; CCI, comprehensive complication index.P value of less than 0.05 was considered statistically significant.

In patients who had C-D classification Grade III or higher, 8 cases of anastomotic fistula, 3 cases of ileus, 6 cases of wound dehiscence, 11 cases of postoperative infection, 1 case of acute myocardial infarction, 4 cases of active hemorrhage, 1 case of delayed gastric emptying, 1 case of subcutaneous abscess, 1 case of arrhythmia, and 11 cases of persistent air leaks in the lungs, 2 cases of chest chyle fistula, 1 case of hypoxemia, 1 case of heart failure, 1 case of ARDS, 1 case of dysuresia occurred. A total of three patients experienced postoperative complications of Grade V in C-D classification, postoperative death, and the causes of death were postoperative infection, acute cardiac infarction and ARDS, respectively. (See **Table 4** for specific classifications).

**Table 4:** Cases of C-D classification Grade III or higher.

Complications	Number	IIIa	IIIb	IVa	IVb	V	Proportion of all,%
Anastomotic fistula	8	4	3		1		0.29
Ileus	3	3					0.11
Wound dehiscence	6	2	4				0.22
Postoperative infection	11	2	2	1	5	1	0.40
Acute myocardial infarction	1					1	0.04
Active hemorrhage	4	2	1		1		0.14
Delayed gastric emptying	1	1					0.04
subcutaneous abscess	1	1					0.04
arrhythmia	1	1					0.04
Persistent air leaks in the lungs	11	8	2	1			0.40
Chest chyle fistula	2		2				0.07
Hypoxemia	1			1			0.04
Heart failure	1				1		0.04
ARDS	1					1	0.04
Dysuresia	1	1					0.04

One patient may have a combination of complications.

ARDS, Acute respiratory distress syndrome.

### Effects of regional blocks on postoperative complications graded by higher C-D classification

As shown in **Table 3**, among patients with C-D classification Grade II or higher complications, regional blocks were used in 462 (22.7%), (Odds ratio (OR) 0.714, 95% Confidence interval (CI) 0.559 to 0.912 (*P*=0.007). Among patients with C-D classification Grade III or higher complications, regional blocks were used in 33 (1.6%), with OR 0.736, 95% CI 0.427 to 1.269 (*P*=0.270). The mean ± SD of CCI in two groups was 14.0 ± 9.7 vs 20.6 ± 11.9, the β value of regional block application was -0.843, 95%CI (-1.629 to -0.056) (*P*=0.036).After matching, in **Table 5**, among patients with C-D classification Grade II or higher complications, regional blocks were used in 216(30.3%) patients, with 348(48.9%) in GA group (OR 0.742, 95% CI 0.552 to 0.996 (*P* =0.047). Among patients with C-D classification Grade III or higher complications, regional blocks were used in 19(2.7%), with OR 0.818, 95% CI 0.441 to 1.514 (*P*=0.522). The mean ± SD of CCI in two groups was 16.2 ± 11.9 vs 20.2 ± 11.7, the β value of regional block application was -0.651, 95%CI (-1.694 to 0.392) (*P*=0.221).

**Table 5:** Sensitivity and adjusted analyses.

Unmatched				
Outcome variables*	OR	*P* value	Lower 95%	Upper 95%
≥Grade II	0.714	**0.007**	0.559	0.912
≥Grade III	0.736	0.270	0.427	1.269
Outcome variable†	Unstandardized Coefficients B	*P* value	Lower 95%	Upper 95%
CCI (%)	-0.843	**0.036**	-1.629	-0.056
postoperative length of stay	-0.371	0.133	-0.856	0.113
Total cost^#^	-0.399	0.397	-1.322	0.524
Matched				
Outcome variables*	OR	*P* value	Lower 95%	Upper 95%
≥Grade II	0.742	**0.047**	0.552	0.996
≥Grade III	0.818	0.522	0.441	1.514
Outcome variable†	Unstandardized Coefficients B	*P* value	Lower 95%	Upper 95%
CCI (%)	-0.651	0.221	-1.694	0.392
postoperative length of stay	-0.331	0.374	-1.061	0.399
Total cost^#^	-0.184	0.809	-1.679	1.310

*Multivariable logistic regression was used.

†Multivariable generalized linear modeling was used.

^#^Ten thousand RMB.P value of less than 0.05 was considered statistically significant.

### LOS and cost

After matching, there were no noteworthy variations between the two groups regarding the postoperative LOS and total cost incurred during hospitalization, with 6 ± 4 vs 8 ± 9(*P*=0.374) in postoperative LOS and 8.6 ± 4.7 vs 9.9 ± 18.4(*P*=0.809) in total cost incurred during hospitalization.

Besides, the incidence of C-D classification Grade II/III or higher complications and CCI was strongly associated with postoperative hospitalization days before or after matching(*P*<0.001).

## Discussion

Postoperative outcomes in elderly patients with cancer are often worrisome. The prevention of postoperative complications in elderly patients with cancer is a challenging and critical task because of co-existing diseases and concurrent medications, diminished functional status and physiological reserve and age-related pharmacodynamic and pharmacokinetic changes in elderly patients. Undoubtedly, PNBs improve analgesia efficacy and reduce opioid requirements and their side effects. The present study evaluated the occurrence of postoperative complications in elderly patients with cancer receiving a PNB in the real world and demonstrated that the utilization of PNBs reduced high-grade complications graded by C-D classification.

Population aging, resulting from the decline in fertility rates and increased life expectancy, has significant social and economic impacts on the world. By 2030, 1 in 6 people in the world will be aged 60 years or above (20). At the same time, the number of elderly cancer patients is increasing year by year, often with one or two underlying diseases and a worrisome health condition. Despite great progress in the care of older surgical patients, they remain more likely to have more postoperative complications, extended length of hospital stays, and higher healthcare costs than younger counterparts (21, 22). Numerous studies have shown high operative mortality in older patients who underwent emergency procedures (10, 23). Although we did not have as large a volume of data and were a single-center study, we included all relevant postoperative complications requiring action and graded them according to the C-D classification, demonstrating that regional blocks do reduce postoperative interventions for patients, reduce medical consumption, and decrease length of stay. To systematic measurement of whether regional blocks provide benefit to older patients, we assessed the effects of PNBs on postoperative complications in elderly patients with cancer undergoing radical colorectal resection, thoracoscopic pneumonectomy. By using the C-D classification to rank postoperative complications, we found that the patients receiving a regional block were less likely to experience postoperative complications of Grade II or above. To further verify it, we also calculated CCI to estimate the burden of complications more accurately as only the most serious complication was considered for grading when using the C-D classification system. The CCI has been widely validated for grading the severity of complications and predicting postoperative outcomes in elderly surgical patients (24–26). However, only significant differences of CCI between the two groups before matching were observed.

In addition, we observed postoperative LOS and total cost during the hospitalization. A shorter stay can reduce the cost per discharge and shift care from inpatient to less expensive settings, indicating better efficiency of hospital management (27). In the study, there was not a significant effect of the application of regional block on postoperative LOS or total cost but a marked reduction in RB group, which may be related to the type of surgery and other confounding factors. Elderly patients with cancer have more comorbidities, a poor basic state, and are more easier affected by surgery than normal adults. Among the different types of surgeries, regional blocks can be an effective means of reducing the incidence of postoperative complications and the LOS. Furthermore, individualized care and rapid recovery programs are necessary to reduce mortality rates. Although training in regional anesthetics has increased, the use of PNB in patients requires more experience and modifications in local anesthetic concentrations, adjuvants, and infusions (28). Of course, large-scale real world studies are needed to verify the effects of regional block in elderly patients with cancer.

It is noteworthy that the single-center study may have a certain bias despite we used real data to illustrate the benefits of regional block in elderly patients. First, considering the type of surgery and the application of nerve blocks the type of surgery was restricted to radical colorectal resection and thoracoscopic pneumonectomy. We studied an elderly population, so we chose the two surgeries in which postoperative complications had a greater impact on survival, however, it may increase the incidence of serious postoperative complications and produce bias in the results. Second, the use of regional block was inevitably influenced by the patient, anesthesiologist, timing of surgery, surgeons, and other subjective factors (29). Moreover, long-term outcomes in older patients also awaits further investigations, such as readmission and mortality rates of patients at 3 months, 6 months and even 1 year after surgery.

## Conclusion

In summary, the results indicate that the utilization of regional block is a promising approach to enhance postoperative outcomes in elderly patients with thoracic and abdominal cancer, as it effectively reduces the incidence of high-risk complications graded by Clavien-Dindo classification. The center has a standardized algorithm for regional block, which has been shown to benefit older patients undergoing thoracic and abdominal surgery. However, an advantage of regional block in reducing CCI and saving hospital stay were not observed. To achieve optimal results, it is crucial to utilize appropriate regional block techniques and to provide individualized treatment plans based on the patient’s specific needs.

## Data availability statement

The original contributions presented in the study are included in the article/supplementary material, further inquiries can be directed to the corresponding author/s.

## Ethics statement

This study was conducted in accordance with the Declaration of Helsinki at Peking University People’s Hospital between 1 January 2018 and 31 March 2022. Ethical approval for this study (Approval No. 2022PHB159-001) was provided by the Medical Ethics Committee of Peking University People’s Hospital, Beijing, China (Chairperson: Dr. Xueguang Zhu) in 2022.

## Author contributions

WD: Formal Analysis, Methodology, Writing – original draft. YZ: Data curation, Writing – review & editing. HL: Formal Analysis, Writing – review & editing. TZ: Data curation, Writing – review & editing. WZ: Data curation, Writing – review & editing. YF: Methodology, Writing – review & editing. HA: Visualization, Writing – review & editing.
